# Study on the Differentiation of Microbial Communities Under Different Soil Types in the Typical Black Soil Area of Songnen Plain

**DOI:** 10.3390/microorganisms14092045

**Published:** 2026-09-14

**Authors:** Xinyi Wang, Junbo Yu, Ke Yang, Yangyang Chen, Kaiming Wang, Zhiwei Yang, Shaozhong Qiao, Jiayu Wang, Xue Liu, Jiacheng Liu, Chenchen Wang

**Affiliations:** 1 Harbin Natural Resources Survey, China Geological Survey, Harbin 150086, China; wangxinyi@mail.cgs.gov.cn (X.W.); yke@mail.cgs.gov.cn (K.Y.); chenyangyang@mail.cgs.gov.cn (Y.C.); wangkaiming@mail.cgs.gov.cn (K.W.); yangzhiwei@mail.cgs.gov.cn (Z.Y.); qiaoshaozhong@mail.cgs.gov.cn (S.Q.); wangjiayu@mail.cgs.gov.cn (J.W.); liuxue@mail.cgs.gov.cn (X.L.); liujiacheng@mail.cgs.gov.cn (J.L.); wangchenchen@mail.cgs.gov.cn (C.W.); 2 Observation and Research Station of Earth Critical Zone in Black Soil, Ministry of Natural Resources, Harbin 150086, China; 3 College of Geo-Exploration Science and Technology, Jilin University, Changchun 130026, China; 4 Institute of Geophysical and Geochemical Exploration, Chinese Academy of Geological Sciences, Langfang 065000, China

**Keywords:** Songnen Plain, black soil, microbial community, ecological strategy, r/K selection, redundancy analysis

## Abstract

Soil type imposes multilayered environmental filtering via physical structure, chemical properties, and resource availability, shaping microbial community assembly. Using 70 samples (black soil *n* = 27, meadow soil *n* = 32, and brown soil *n* = 11) from a typical black soil region of the Songnen Plain, we compared brown soil (forestland) with black soil and meadow soil (cropland) via 16S rRNA V3–V4 (primers 338F/806R) and fungal (ITS1, primers ITS1F/ITS2) amplicon sequencing and physicochemical analyses. The results showed (1) significantly higher organic carbon in brown soil than in black and meadow soils (*p* < 0.05); (2) a systematic shift along the r–K strategy axis, with K-strategists (e.g., *Mortierellomycota* and *Acidobacteriota*) dominating in brown soil, specializing in recalcitrant organic matter decomposition and carbon sequestration, and r-strategists (e.g., *Proteobacteria*, *Bacteroidota*, and *Sordariaceae*) enriched in black and meadow soils, rapidly utilizing labile carbon and driving carbon release. Redundancy analysis identified total organic carbon and pH as key environmental filters driving this shift. Our findings provide an ecological-strategy perspective for understanding land-use effects on soil microbiomes, informing carbon restoration and sustainable management in black soil regions.

## 1. Introduction

As one of the most abundant biodiversity groups on Earth, soil microorganisms are the core drivers of biogeochemical cycles and regulate key ecological processes such as organic matter decomposition, nutrient transformation, and greenhouse gas emissions [1,2,3]. The soil environment generally shapes the community composition [4], diversity [5], interaction patterns [6], and functional structure of soil bacteria [7,8]. Analyzing the regulation mechanisms of microbial community structure and function has important theoretical value for predicting ecosystem response patterns and maintaining ecosystem service functions. In the study of microbial ecology, the theory of ecological strategy (especially the dichotomy of r-strategy and K-strategy) provides an important paradigm for understanding the mechanism of community adaptation [9,10]. Nutrient-rich (r-strategist) microorganisms are characterized by rapid growth, high ribosomal copy numbers, and preference for easily degradable carbon sources and are dominant in resource-rich environments. Oligotrophic (K-strategist) microorganisms show slow growth and high resource utilization efficiency, and win in the oligotrophic competitive environment by virtue of their ability to degrade complex organic matter [11,12]. This theory provides a key basis for predicting the response of microorganisms to environmental changes. In recent years, the r–K ecological strategy framework has gained increasing application in microbial community studies within black soil regions. Li et al. investigated bacterial communities across a mean annual temperature gradient (0.6–7.3 °C) in northeast China and found that rising temperatures drove a shift from K- to r-strategists, with the dominance of r-strategists promoting rapid organic matter decomposition [13]. Similarly, Fang et al., based on a 44-year long-term field experiment in the same region, reported that fertilization increased the ratio of copiotrophic (r-strategy)-to-oligotrophic (K-strategy) bacterial groups [14]. Furthermore, long-term fertilization significantly influenced both the abundance and ecophysiological functions of r- and K-strategist communities (including bacteria and fungi) in cultivated black soils [15]. Collectively, these findings underscore the applicability of the r–K framework for elucidating microbial community responses to environmental changes and management practices in black soil ecosystems.

In the Hailun area, different soil types are strongly coupled with their land-use history: brown soil is mainly developed in forest-covered hilly slopes and is in a natural vegetation state for a long time, while black soil and meadow soil are distributed in flat plain areas, and most of them have been reclaimed as farmland (maize–soybean rotation). Therefore, the comparison between soil types essentially reflects the difference in land-use history. These three soil types correspond to different land-use patterns, and differences in land-use patterns represent one of the most intense human disturbances to the ecosystem [16]. Black soil and meadow soil used as farmland, due to their tillage disturbance, fertilization input, and continuous monocropping, form a “eutrophication screening environment” with high disturbance and concentrated resource inputs [17,18]. In forestland, brown soil is characterized by stable microenvironment and continuous litter input (rich in refractory organic matter), which constitutes a typical “oligotrophic screening environment” [19]. Therefore, different soil types form a differentiated screening environment by changing the resource supply intensity and disturbance frequency, and then drive the soil microbial community to differentiate towards “eutrophic” or “oligotrophic” ecological strategies.

An important commodity grain base in China and one of the typical black soil belts in the world, the black soil area of the Songnen Plain has experienced large-scale agricultural reclamation in the last century, resulting in the destruction of original vegetation, a sharp decrease in soil organic matter, and the degradation of structure, which pose serious threats to food security and ecological stability [11,20,21,22]. Although studies have confirmed that different soils change the soil microbial community structure [23,24,25], the ecological selection mechanism behind these changes remains to be determined [26]. If the framework is applicable to the differentiation of microorganisms in different soil types in Hailun City, the brown soil forestland should be enriched with oligotrophic microorganisms, while the cultivated land of black soil and meadow soil should be enriched with nutrient-rich microorganisms. At the same time, the key environmental factors that drive this expected differentiation are still unclear. The roles and relative contributions of soil TOC, TN, and pH still need to be quantitatively analyzed. These two problems have not been systematically tested in the typical soil types in the black soil area of the Songnen Plain.

Therefore, in this study, three representative soil types (brown soil, black soil, and meadow soil) in Hailun City were selected as the research objects, and 70 surface soil samples were systematically collected on a regional scale. Illumina MiSeg high-throughput sequencing technology was used to analyze the composition and diversity of bacterial and fungal communities. Combined with soil physical and chemical analysis and multivariate statistical methods, the purpose of this study was to test the above hypotheses and provide a theoretical basis and data support for sustainable soil management under different land-use backgrounds in black soil areas from the perspective of microbial ecology.

## 2. Materials and Methods

### 2.1. Overview of the Study Area and Sample Collection

**Figure 1:**
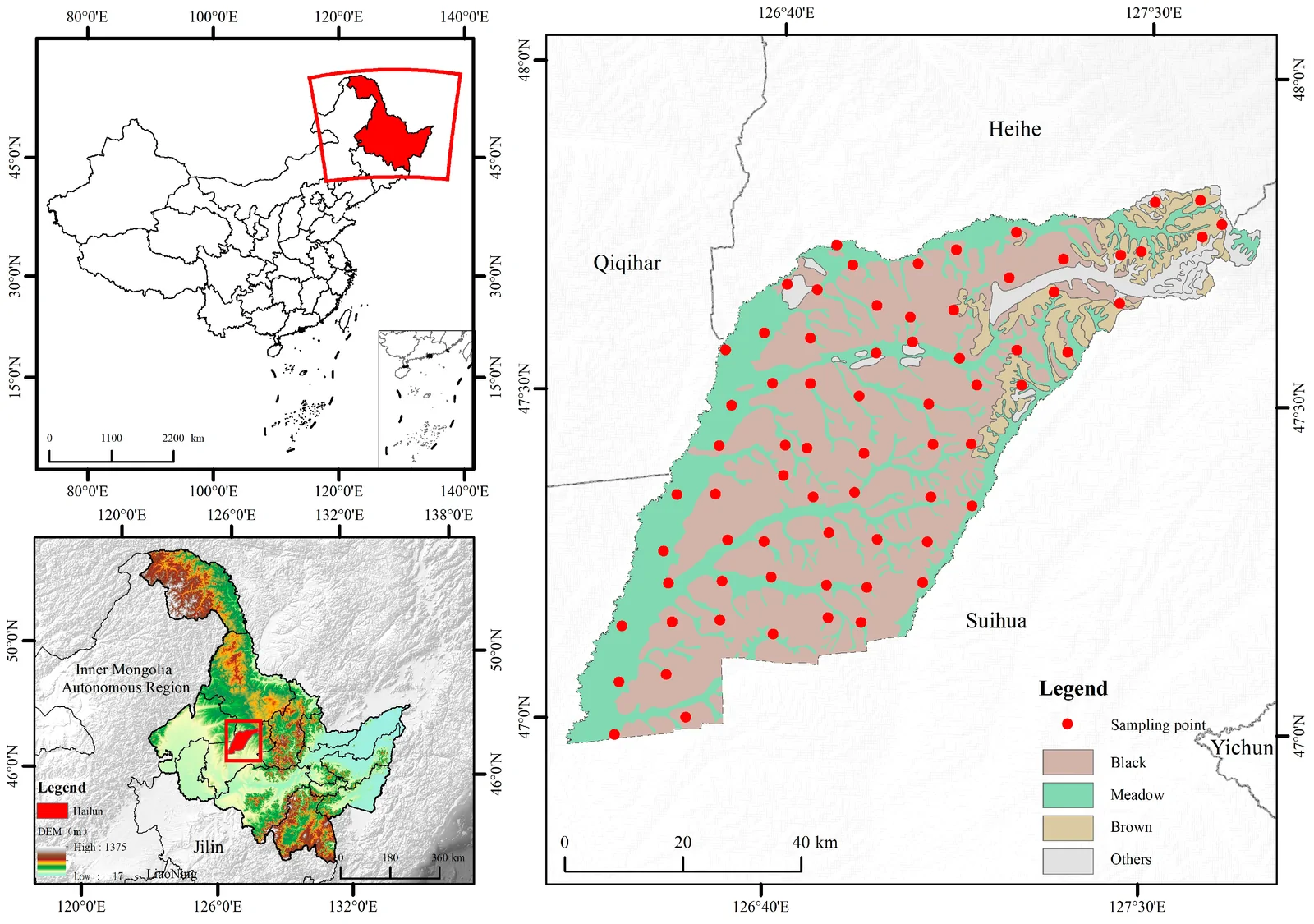
The figure shows the location of Hailun City in the Songnen Plain and the spatial distribution of sampling points.

The study area is located in Hailun City, Heilongjiang Province, China (46°58′–47°52′ N, 126°14′–127°45′ E). It is located in the northeast of the Songnen Plain and is a typical black soil core distribution area (Figure 1). The area has a cold temperate continental climate, with an annual average temperature of 3.4 °C and annual precipitation of 511.2 mm; rainfall and heat occur in the same season, and the precipitation is mainly concentrated in July–August. Three representative soils in Hailun City were selected: Black soil is located on the top of high plains and flood plains, with good drainage. It was originally meadow steppe and has now been reclaimed as farmland. Meadow soil is located at the back edge of low plains and river terraces, with low and flat terrain, seasonal waterlogging, and salinization; it is mostly cultivated as dry land. Brown soil occurs on hilly slopes, with shallow soil and gravel; it is not suitable for farming and has long been forestland.

The layout of sampling points in this study followed the principles of representativeness, randomness, and systematic sampling. Firstly, based on the soil type map of the study area, the area was divided into three soil type units: black soil, meadow soil, and brown soil. The stratified random sampling method was used to lay out the sampling points, and the random points were fine-tuned considering factors such as topography, land-use status, and accessibility to ensure that the sampling points could represent the main characteristics of each soil type. Field samples were collected in July 2023. A total of 70 surface soil (0–20 cm) samples were collected, covering the above three soil types (black soil *n* = 27, meadow soil *n* = 32, and brown soil *n* = 11). About 1 kg of soil was collected by the three-point mixing method at each sampling point, and the soil was fully mixed after removing stones and plant roots. The samples were divided into two parts: one part was immediately placed in a foam box containing dry ice, transported back to the laboratory, and stored in an ultralow-temperature refrigerator at −80 °C for microbial genome sequencing analysis; the other part was dried indoors for the determination of soil physical and chemical properties.

### 2.2. Measurements of Soil Properties

Soil samples for physical and chemical analysis were sifted through a 2 mm nylon sieve (Shanghai Shupei Laboratory Equipment Co., Ltd., Shanghai, China). Some sieved soil samples were further ground to 0.15 mm for total organic carbon (TOC) and total nitrogen (TN) analysis. Three technical replicates were performed for all measurements. Soil pH and electrical conductivity (EC) were determined according to HJ 962-2018 and HJ 802-2016, respectively, using a pH meter and a conductivity meter (both from Shanghai INESA Scientific Instrument Co., Ltd., Shanghai, China). Soil Na^+^ concentration was determined by flame photometry using the acid digestion solution, according to NY/T 296-1995. Soil K^+^ concentration was determined by flame photometry using the acid digestion solution, according to NY/T 87-1988. The concentration of anion Cl^−^ was determined by silver nitrate titration according to NY/T 1378-2007. Soil TOC content was determined by the combustion oxidation–titration method according to HJ 658-2013. Soil TN content was determined by the modified Kjeldahl method according to HJ 717-2014.

### 2.3. Microbial DNA Extraction and High-Throughput Sequencing

Total genomic DNA was extracted from 0.5 g of each soil sample. The DNA concentration and purity were determined using a NanoDrop 2000 spectrophotometer (Thermo Fisher Scientific, Wilmington, DE, USA), and DNA integrity was assessed by 1.2% (*w*/*v*) agarose gel electrophoresis.

The V4–V5 hypervariable region of the bacterial 16S rRNA gene [27] was amplified with the primer pair 338F (5′-ACTCCTACGGGAGGCAGCAG-3′) and 806R (5′-GGACTACHVGGGTWTCTAAT-3′) [28]. For fungi, the ITS1 region was amplified using the primer set ITS1F (5′-CTTGGTCATTTAGAGGAAGTAA-3′) and ITS2 (5′-GCTGCGTTCTTCATCGATGC-3′) [29]. Each PCR reaction mixture contained 15 µL of Phusion High-Fidelity PCR Master Mix, 0.2 µM of each primer, and 10 ng of genomic DNA template. The thermal cycling program consisted of an initial denaturation at 98 °C for 1 min, followed by 30 cycles of denaturation at 98 °C for 10 s, annealing at 50 °C for 30 s, and extension at 72 °C for 30 s, with a final extension step at 72 °C for 5 min. Three technical replicates were performed for each sample. Amplicons from technical replicates of the same sample were pooled in equal amounts and purified using magnetic beads. A blank control for DNA extraction and a PCR negative control (ddH_2_O) were included throughout the experiments. Subsequent procedures were only carried out when no visible band was observed in the PCR negative control. Purified amplicons were quantified and pooled at equimolar concentrations for library construction. Library quality was verified by Qubit fluorometry and qPCR, and qualified libraries were subjected to paired-end 300 bp (PE300) sequencing on the Illumina MiSeq platform. To minimize batch effects, amplification and library preparation for all samples were performed in a single batch. Trace reads derived from negative controls (generally <1000 reads) did not interfere with the statistical analysis of differential results. 

### 2.4. Bioinformatics Analysis

The raw reads obtained by sequencing were first subjected to quality control using fastp (v0.20.1) to remove adaptor sequences and low-quality bases. Subsequent processing was performed using the QIIME 2 (v2022.2) process [30].

The paired-end sequence after quality control was denoised and merged, and chimera was removed through the DADA2 plug-in to generate the Amplicon Sequence Variant (ASV) feature table representing the precise biological sequence. Using the classify-sklearn method in the feature-classifier plugin of QIIME 2, the ASV sequences were compared with the pre-trained classifier to obtain the taxonomic annotation. The reference database of bacterial 16S ASV was SILVA (v138), and the reference database of fungal ITS ASV was UNITE (v8.3). In order to reduce the interference of sequencing noise and rare sequences with statistical analysis, mitochondria, chloroplast sequences, and extremely low-abundance ASVs (e.g., ASVs with a total abundance of less than 10 or in less than 2 samples) were removed from the ASV table.

### 2.5. Data Processing and Analysis

The geographic location of the sample was recorded by Beidou. The Kruskal–Wallis test was used to analyze differentially abundant taxa at the phylum and order levels, and taxa with relative abundance greater than 0.1% were counted at these levels. The species accumulation curve and diversity index were calculated and plotted using the Vegan package in R v3.53 and Rstudio v1.1.463. At the order level, R v3.53 and RStudio (v1.1.463) software (Pheatmap) with the pheatmap package were used to draw the clustering heatmap. Redundancy analysis (RDA) was performed using Canoco 5 to assess the relationship between species and soil properties for taxa with significant differences at the phylum and order levels.

### 2.6. Classification of Microbial Ecological Strategies

To classify bacterial taxa into ecological strategies, we adopted the r/K selection framework proposed by Fierer et al. Based on the known life-history traits and ecological preferences of each bacterial phylum, taxa were categorized as putative K-strategists (oligotrophs, e.g., Acidobacteria, *Gemmatimonadetes*, and *Verrucomicrobia*) or r-strategists (copiotrophs, e.g., Proteobacteria, *Bacteroidetes*, and *Firmicutes*). This classification scheme was applied consistently across all samples [31].

For fungal communities, we followed the method described by Yang et al. to classify taxa at the phylum level into putative r-strategists and K-strategists. Accordingly, *Ascomycota* and *Zygomycota* were assigned as r-strategists, whereas *Basidiomycota* were assigned as K-strategists [32]. Additionally, following forest succession-related studies, *Rozellomycota* were also classified as r-strategists, and *Mortierellomycota* as K-strategists [33].

Taxa that were not clearly assigned in the literature were conservatively excluded from the r/K strategy-specific analyses.

## 3. Results

### 3.1. Soil Physical and Chemical Properties Under Different Soil Types

The analysis of soil physical and chemical properties showed that, except for TOC and TN, the three soil types did not differ in other physical and chemical characteristics (such as pH and conductivity) (Table 1). The contents of total organic carbon (mean 3.35 g/kg) and total nitrogen (mean 3.78 g/kg) in brown soil were significantly higher than those in black soil and meadow soil, reflecting the characteristics of long-term organic matter accumulation in the forest ecosystem. The pH value of brown soil (5.75 on average) was slightly lower than that of black soil (5.98 on average) and meadow soil (6.19 on average), and the conductivity of all samples was low (0.03–0.19 mS/cm), indicating that there was no obvious salinization in the study area. The results showed that long-term forestland cover and agricultural tillage mainly changed soil carbon and nitrogen storage capacity (TOC and TN) but had little effect on basic physical and chemical properties such as soil pH and salt (conductivity). The high carbon and nitrogen contents of brown soil confirmed the significant shaping effect of long-term organic matter accumulation on soil nutrient pool.

### 3.2. α and β Diversity of Microbial Community

**Figure 2:**
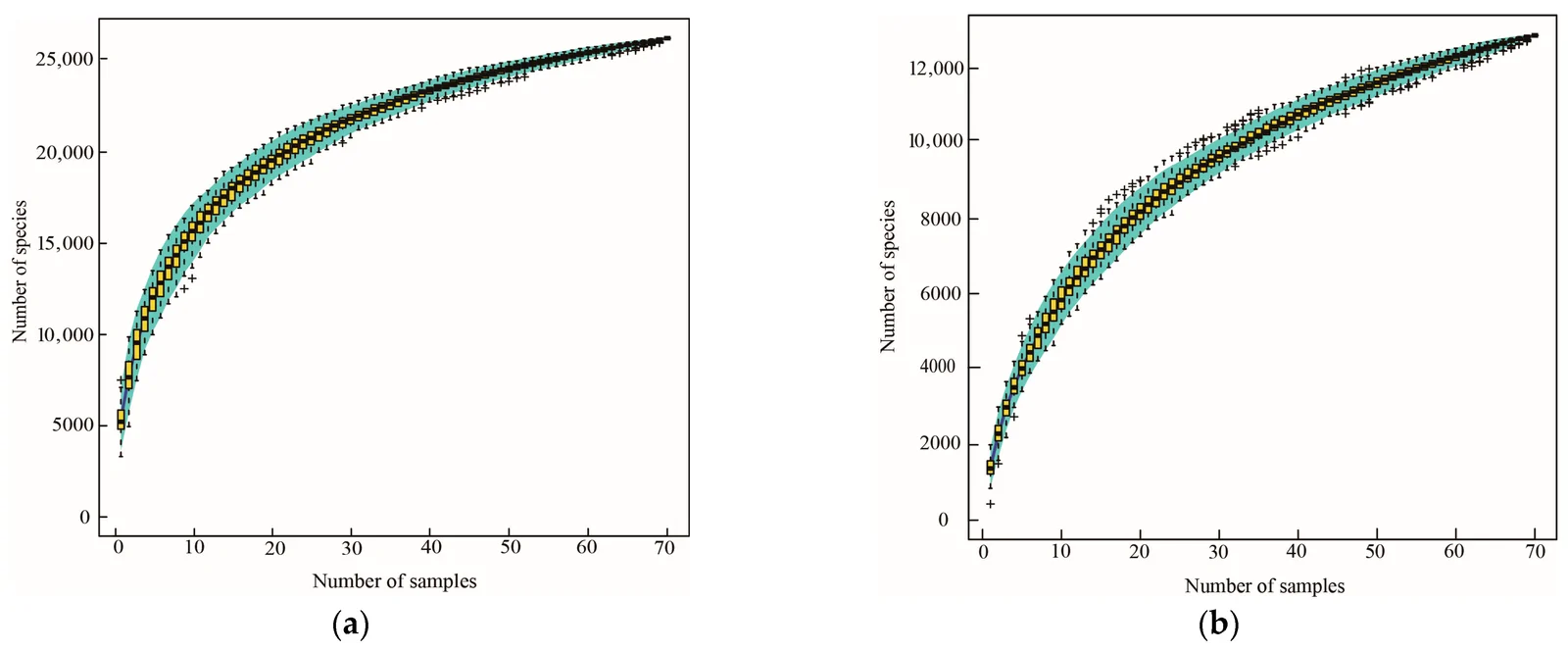
Cumulative curves of bacterial (**a**) and fungal (**b**) species. The abscissa represents the number of randomly selected samples (*n*), and the ordinate represents the cumulative total number of microbial ASVs detected under the corresponding sample number. The yellow squares indicate the mean cumulative number of species, the black crosses indicate individual random permutations, and the blue shaded band indicates the 95% confidence interval.

After quality control and filtration, a total of 4,418,431 high-quality bacterial sequences and 5,693,236 high-quality fungal sequences were obtained from 70 soil samples by high-throughput sequencing of the 16S rRNA gene and the ITS region. The species accumulation curve at the ASV level (Figure 2) exhibited a sustained upward trend with increasing sample size, confirming that the sequencing depth was sufficient to capture community structure variations among samples and to meet the requirements for subsequent analyses.

**Figure 3:**
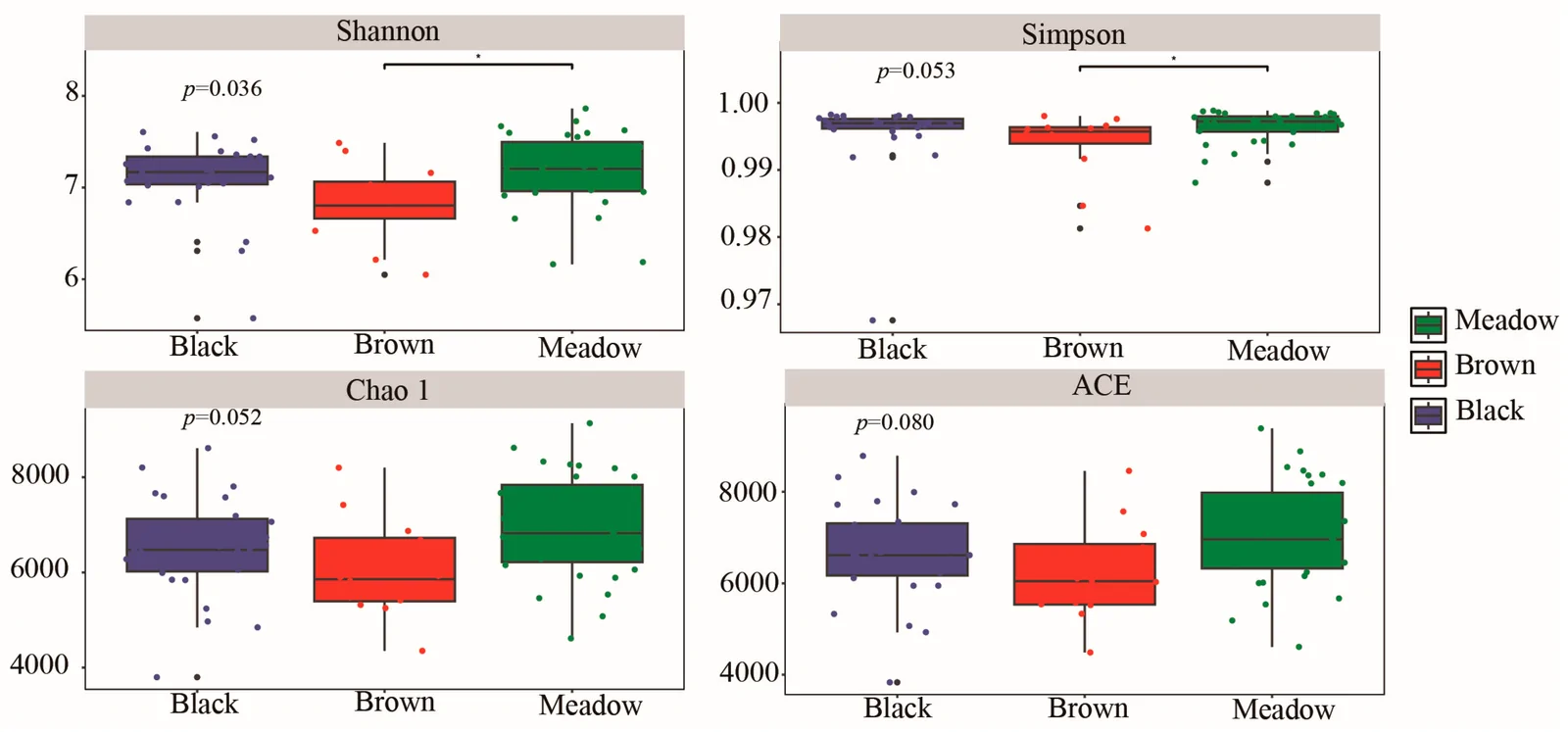
The difference test of bacterial community α diversity in Hailun area. The mean distribution of the Shannon, Simpson, Chao1, and ACE indices is shown in the figure. The asterisks denote significant differences between pairwise comparisons: * *p* < 0.05, ** *p* < 0.01.

**Figure 4:**
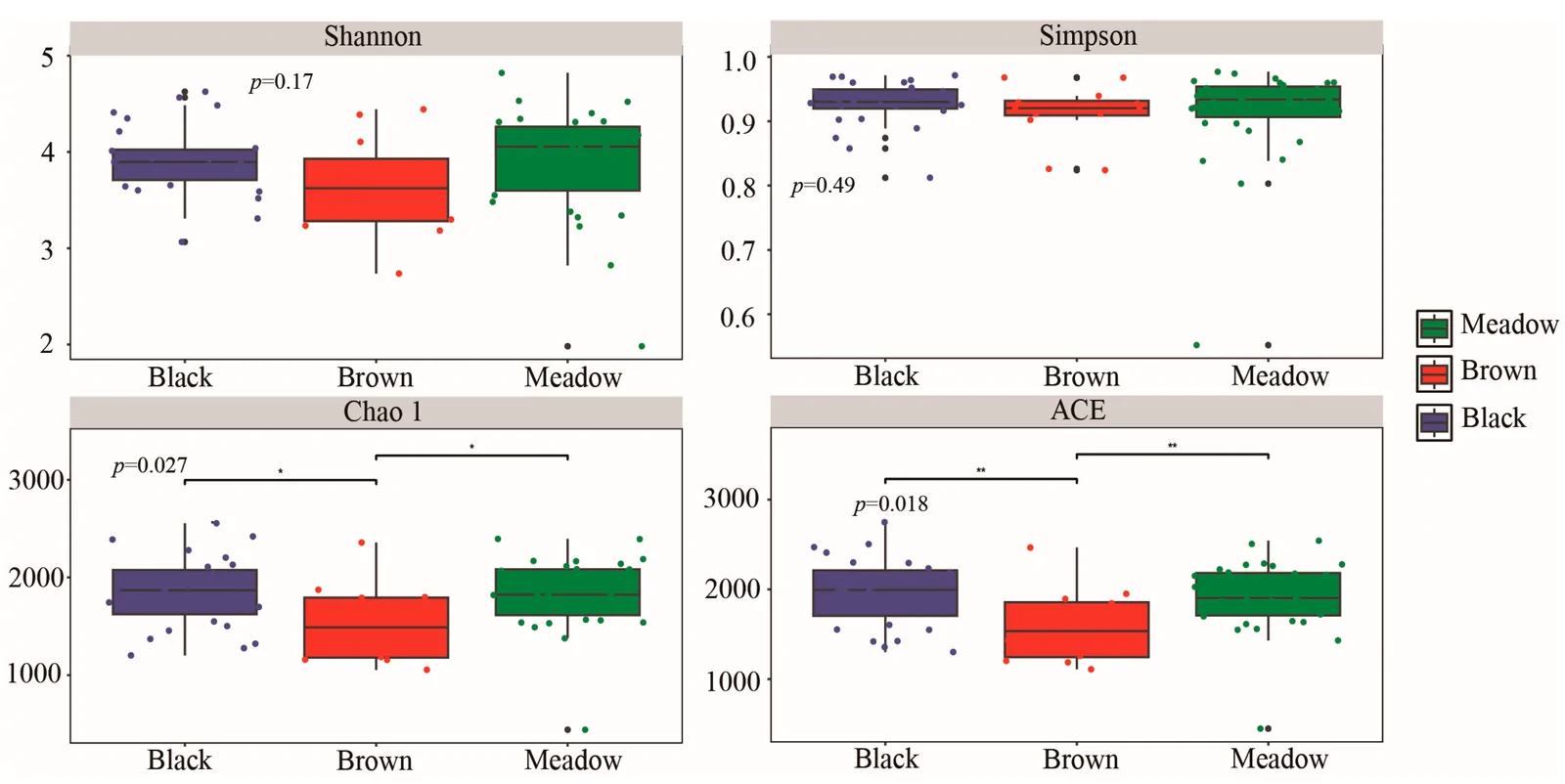
Difference test of fungal community α diversity in Hailun area. The mean distribution of the Shannon, Simpson, Chao1, and ACE indices is shown in the figure. The asterisks denote significant differences between pairwise comparisons: * *p* < 0.05, ** *p* < 0.01.

For α diversity, there were differences in microbial community richness (Chao1 and ACE) and diversity (Shannon and Simpson) indices among different soil types. In the analysis of differences in α diversity indices among groups, the boxplot can intuitively reflect the median, dispersion degree, maximum value, minimum value, and outlier of species diversity in the group. Kruskal–Wallis test and Dunn post hoc multiple comparisons (Bonferroni correction) were used to compare groups. For the bacterial community, the Shannon index (*p* = 0.036) and Chao1 index (*p* = 0.052) of brown soil were lower than those of black soil and meadow soil (Figure 3). For the fungal community, the Chao1 index (*p* = 0.027) and ACE index (*p* = 0.018) of brown soil were significantly lower than those of the other two agricultural soils (Figure 4).

**Figure 5:**
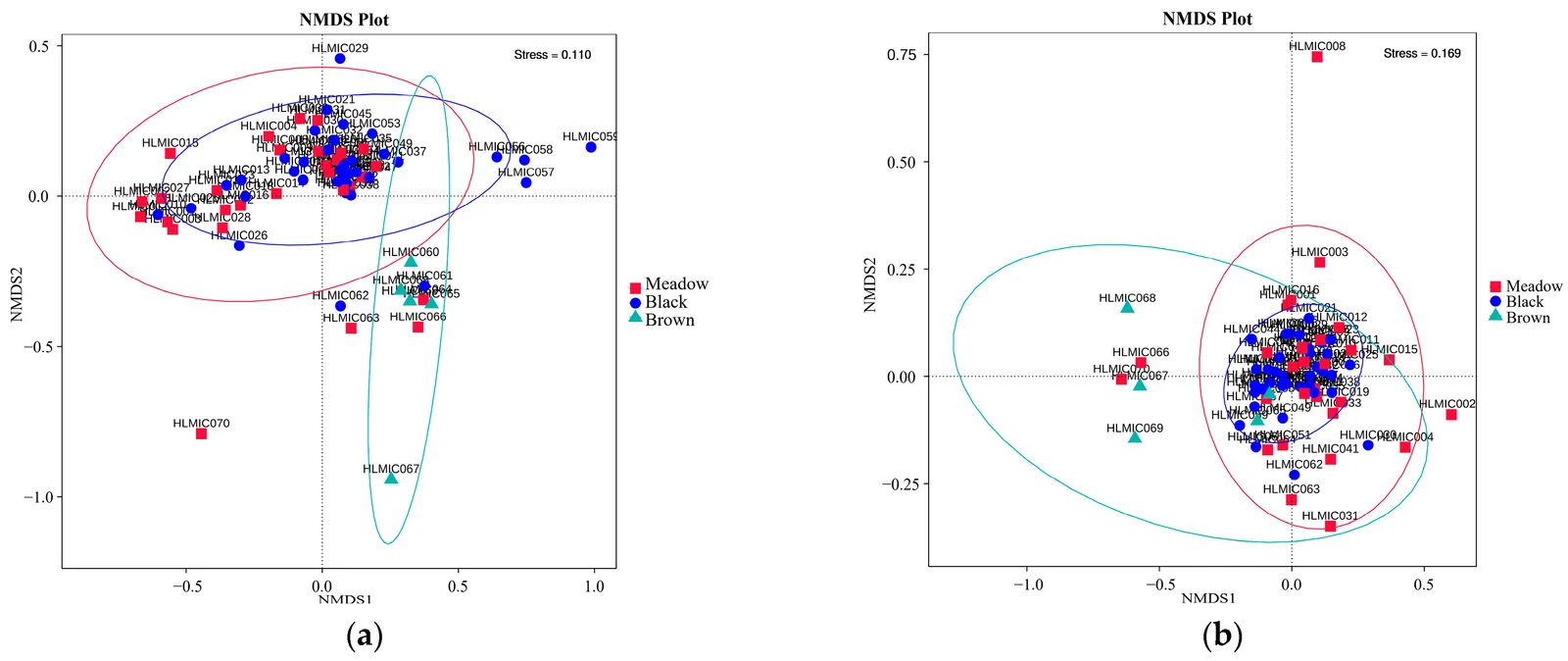
NMDS ordination (Bray–Curtis) of bacterial (**a**) and fungal (**b**) community structures across treatment groups. Stress values: 0.110 (**a**) and 0.169 (**b**), indicating reliable ordination. The dashed line represents the zero-reference line corresponding to NMDS1 = 0 and NMDS2 = 0.

Based on Bray–Curtis dissimilarity, NMDS analysis showed that the microbial communities of the three soil types were clearly separated (Figure 5). The PERMANOVA overall test indicated that the differences in community structure were highly significant (*p* < 0.001); pairwise comparisons showed that brown soil (forest) was significantly separated from black soil and meadow soil (cropland) (*p* < 0.01), while the difference between black soil and meadow soil was not significant (*p* = 0.23), indicating that the microbial communities of the two cropland soils were similar. The PERMDISP test showed no significant differences in within-group dispersion (*p* > 0.05), confirming that the clustering was due to shifts in community centroids rather than within-group variation. In summary, soil type affects land-use type, which in turn is the main factor influencing soil microbial community structure in this region.

### 3.3. Ecological Strategies for Microbial Community Composition

The Kruskal–Wallis sum test (multiple-group comparison) was used to identify order-level taxa whose relative abundances differed significantly among the three soil types (black soil, meadow soil, and dark brown forest soil), with *p* < 0.05 as the threshold for statistical significance. Subsequently, linear discriminant analysis (LDA) was performed to evaluate the contribution of each differentially abundant order to group discrimination, and taxa with an LDA score > 2 were retained as having significant biological relevance (high effect size). In the heatmap, color gradients represent Z-score standardized relative abundances of each order-level taxon, with orange indicating taxa relatively enriched in a given soil type and green indicating those relatively depleted.

**Figure 6:**
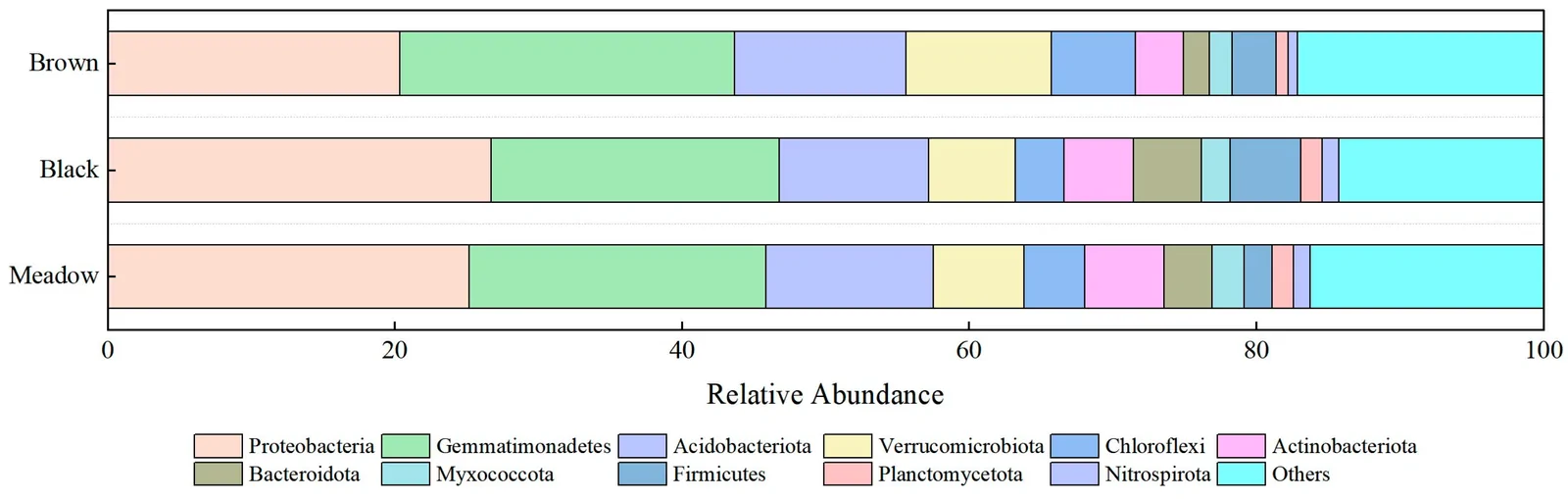
The relative abundance of dominant bacteria under different soil types. The relative abundance of other phyla was less than 1%.

**Figure 7:**
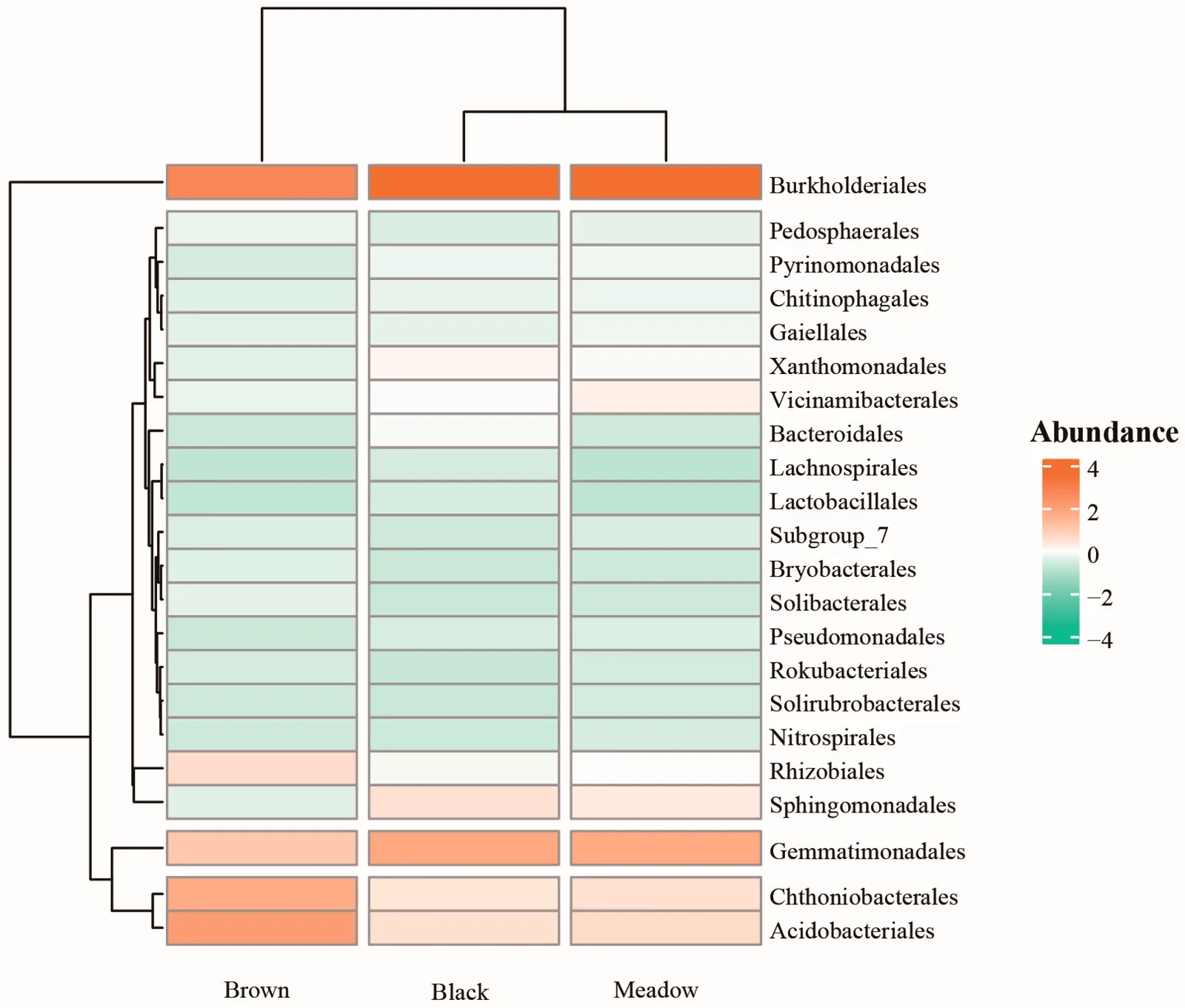
Order-level clustering heatmap of bacterial communities across different types.

As shown in Figure 6 and Figure 7, bacterial community composition exhibited pronounced differentiation among soil types. Within *Proteobacteria*, orders such as *Xanthomonadales* and *Burkholderiales* were relatively more abundant in black soil and meadow soil, suggesting that these taxa may be better adapted to soil environments with high organic matter content or neutral pH. In contrast, *Acidobacteriales* and *Bryobacterales*, belonging to *Acidobacteriota*, were significantly enriched in dark brown forest soil, implying a greater tolerance to low pH or nutrient-stressed conditions. In addition, several other taxa showing significant differences from the phylum to order levels were identified (e.g., *Gemmatimonadales*, *Solibacterales*, and *Subgroup_2*). Their distribution patterns across soil types further corroborated the sensitivity of microbial community structure to soil type. Collectively, these findings indicate that land-use type is a major driver of bacterial community composition and that specific taxa within *Proteobacteria* and *Acidobacteriota* may serve as potential indicator groups for different soil types.

**Figure 8:**
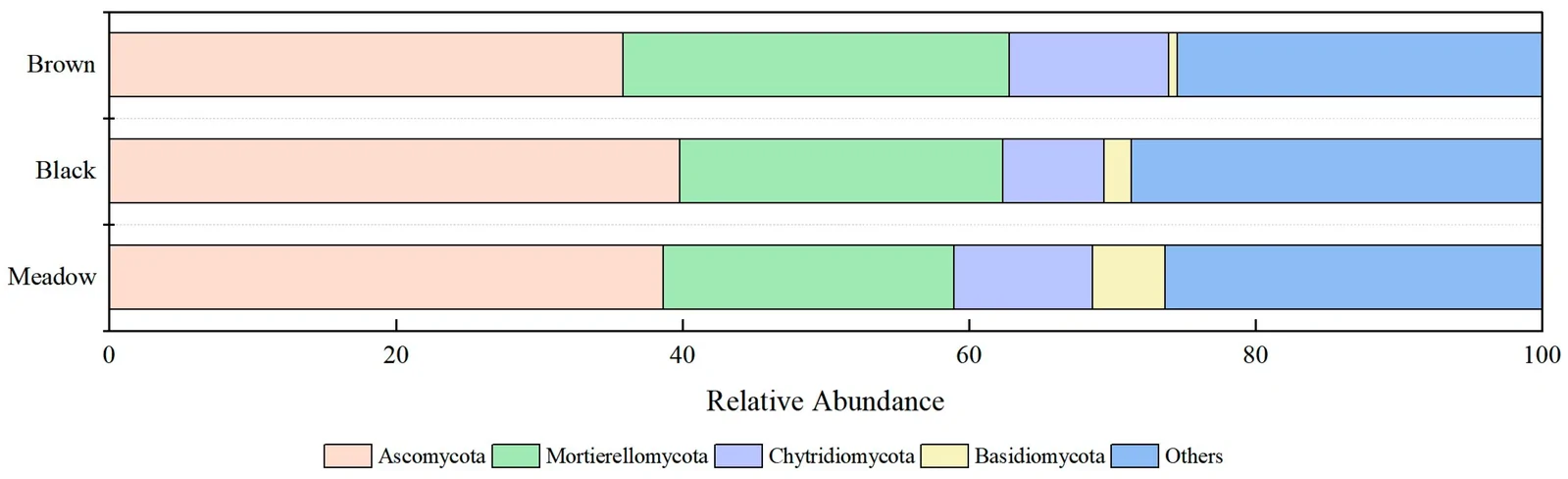
The relative abundance of dominant fungi under different soil types. The relative abundance of other phyla was less than 1%.

**Figure 9:**
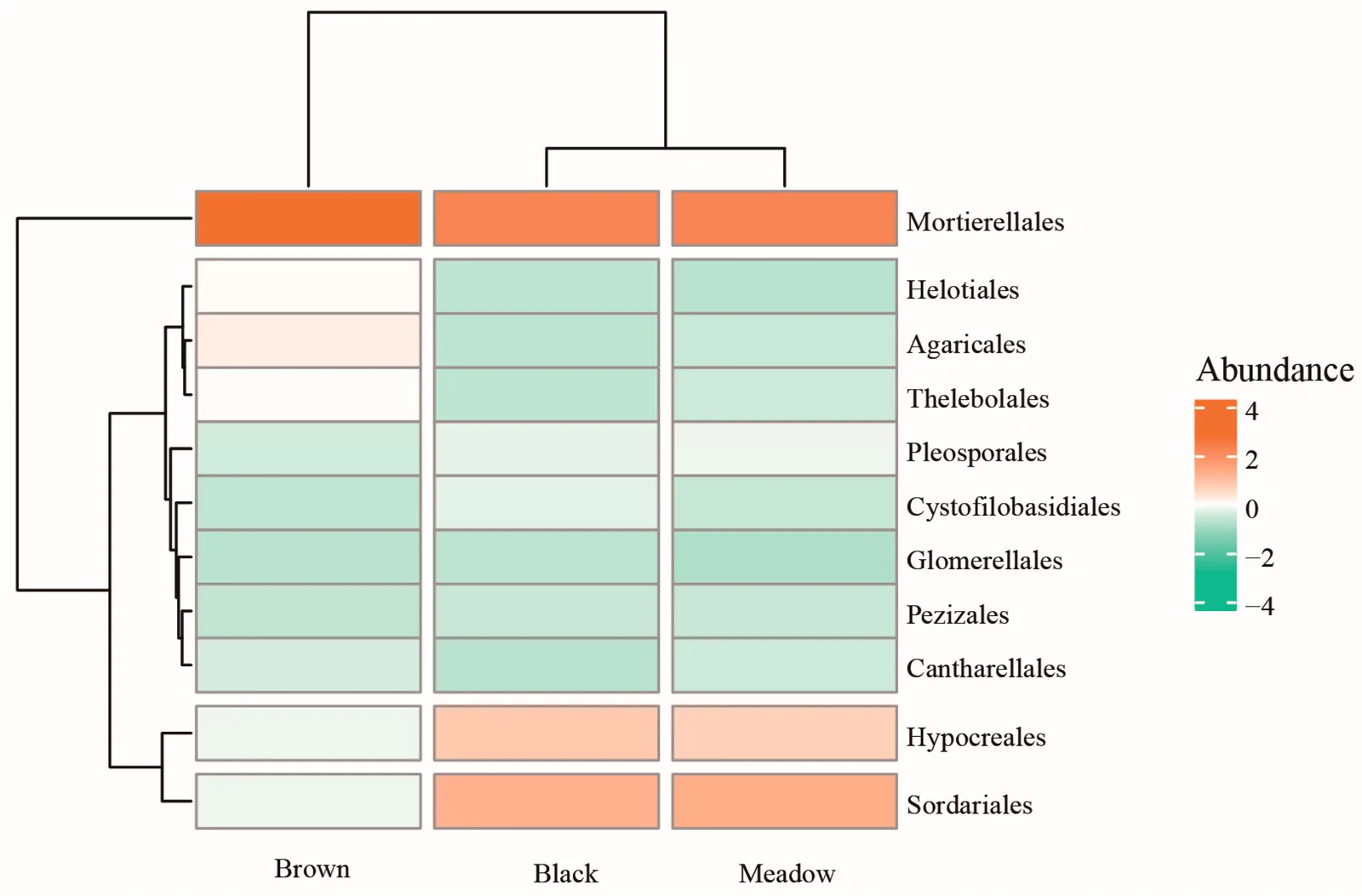
Order-level clustering heatmap of fungal communities across different soil types.

As shown in Figure 8 and Figure 9, fungal community composition also exhibited clear ecological niche differentiation among the three soil types. In meadow soil, *Mortierellales*, *Helotiales,* and *Agaricales* were significantly enriched. *Mortierellales*, as typical saprotrophic fungi, showed a competitive advantage in meadow soil rich in readily decomposable organic matter; *Helotiales* and *Agaricales* are often associated with plant roots or involved in litter decomposition, reflecting the higher root biomass and carbon input that are characteristic of meadow soils. In black soil, *Hypocreales*, *Sordariales*, and *Eurotiales* predominated. These taxa are mostly saprotrophic fungi adapted to high-nutrient conditions, and their enrichment is consistent with the long-term accumulation of organic matter and the high fertility of black soil, suggesting that they could serve as potential bioindicators of fertile soils. In contrast, dark brown forest soil was significantly enriched in mycorrhizal fungal orders such as *Glomerales*, *Thelephorales*, and *Russulales*. Most of these taxa form ectomycorrhizal or arbuscular mycorrhizal symbioses with plants and exhibit greater ecological adaptability under nutrient-limited or acidic soil conditions, implying that dark brown forest soil may possess stable forest soil characteristics and strong mycorrhizal symbiosis potential. In summary, fungal communities at the order level are highly responsive to changes in soil type. Taxa such as *Mortierellales*, *Helotiales*, and *Hypocreales* can be considered potential indicator groups for different soil types. This study demonstrates that LEfSe analysis effectively identified fungal biomarkers for each soil type, providing important insights into the ecological functions of fungal communities across different soil environments.

### 3.4. The Driving Effect of Environmental Factors on Microbial Communities

In order to reflect the correspondence between microorganisms and environmental factors, detrended correspondence analysis (DCA) was first performed at the order level, and the maximum axis length was less than 3. Therefore, redundancy analysis (RDA) was more appropriate for identifying the environmental driving factors affecting the distribution of bacterial communities [34,35]. RDA can effectively reflect most of the information between the soil microbial community and environmental factors. The projection length of the arrow represented by environmental factors on the horizontal axis represents the driving force of environmental factors to the microbial community. The cosine of the angle between arrows representing an environmental factor and the microorganism represents the correlation between them [36,37]. Red represents environmental factors, and black represents microorganisms.

**Figure 10:**
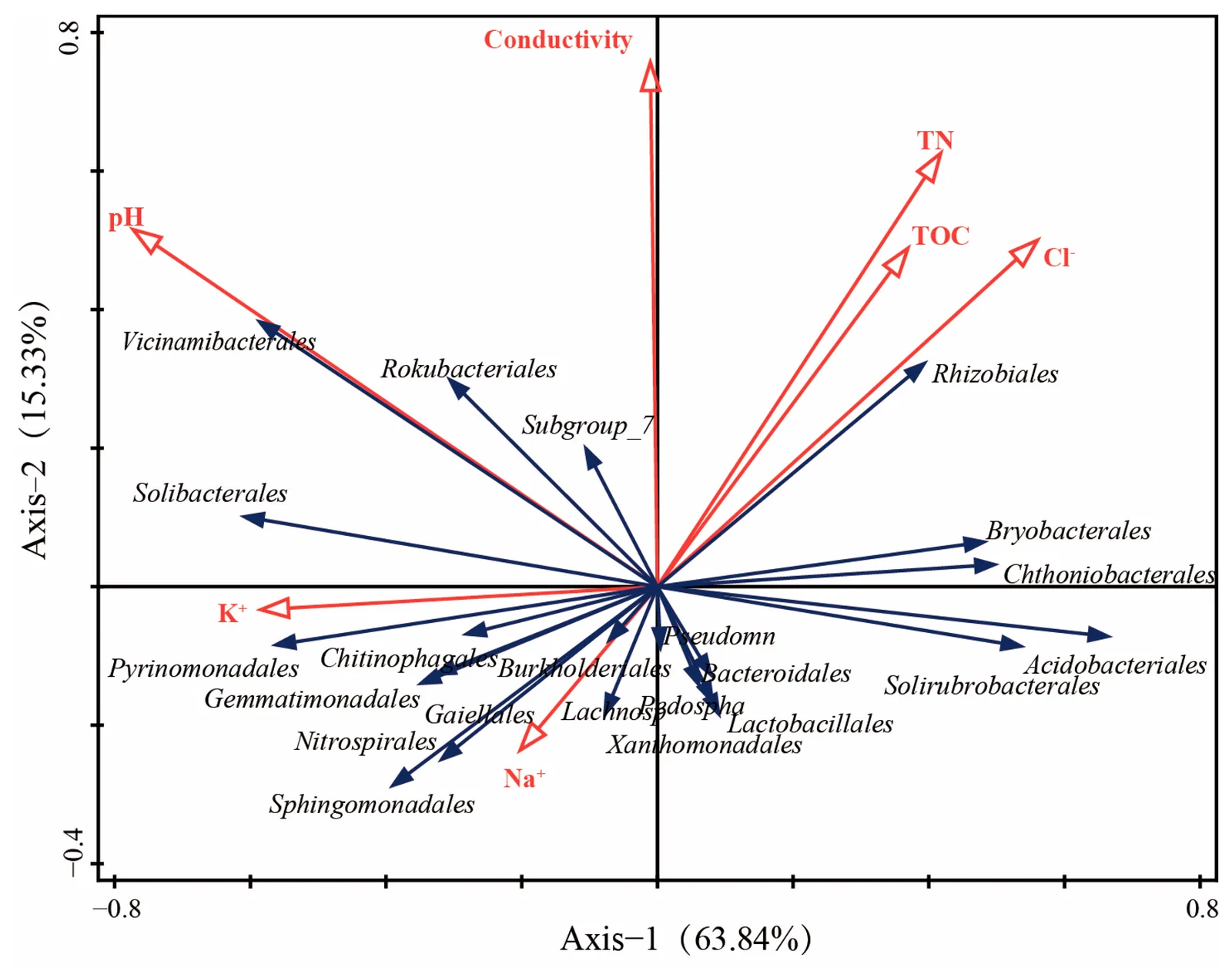
Redundancy analysis of bacterial community at the order level. In the figure, red arrows represent soil environmental factors, and black arrows represent the main bacterial orders.

RDA indicated that environmental factors significantly affected the composition of bacterial communities at the order level in the Hailun area. As shown in Figure 10, the first ordination axis (Axis-1) explained 63.84% of the total variation, and the second ordination axis (Axis-2) explained 15.33%, with a cumulative explanation of 79.17%. This indicates that the selected environmental factors effectively explained the structural variation in the bacterial community. Based on the length and direction of the environmental factor vectors, pH, Cl^−^, TOC, TN, and conductivity were the primary environmental variables driving bacterial community distribution. Specifically, pH was positively correlated with orders such as *Vicinamibacterales* and *Rokubacteriales*, while TOC, TN, and Cl^−^ were closely associated with orders including *Rhizobiales*, *Bryobacterales*, and *Chthoniobacterales*. Additionally, Na^+^ and K^+^ mainly influenced orders such as *Sphingomonadales* and *Nitrospirales*. As verified by 999 Monte Carlo permutation tests, the effects of the main influencing factors (e.g., pH, Cl^−^, and TOC) on bacterial community composition were statistically significant (*p* < 0.05).

**Figure 11:**
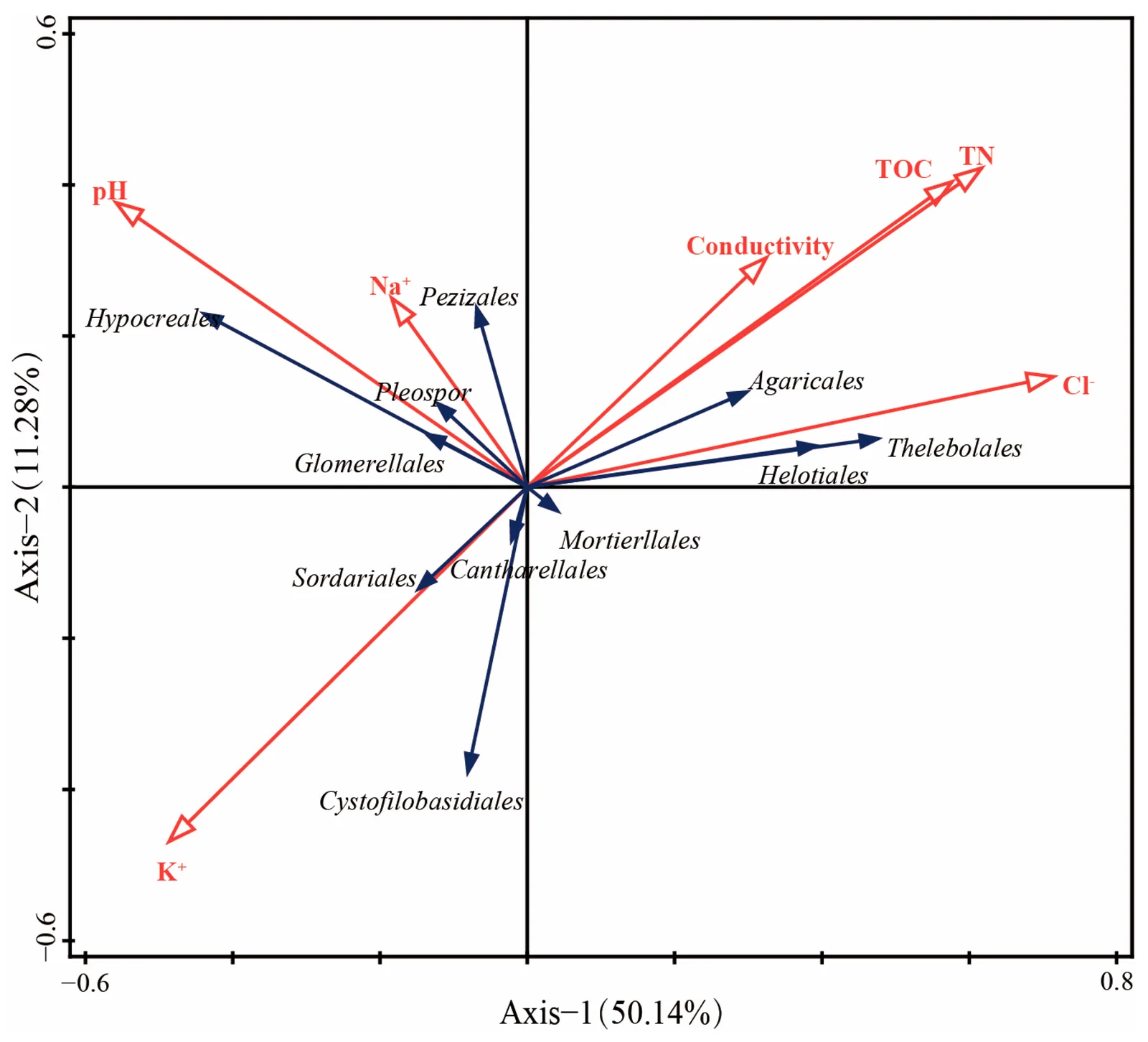
Redundancy analysis of fungal community at the order level. In the figure, red arrows represent soil environmental factors, and black arrows represent the main fungal families.

RDA indicated that environmental factors significantly affected the composition of fungal communities at the order level in the Hailun area. As shown in Figure 11, the first ordination axis (Axis-1) explained 50.14% of the total variation, and the second ordination axis (Axis-2) explained 11.28%, with a cumulative interpretation of 61.42%. This indicates that the selected environmental factors effectively reflected the structural changes in the fungal community. The long vectors of environmental factors including pH, TOC, TN, conductivity, Cl^−^, Na^+^, and K^+^ suggested that they were the primary variables driving fungal community distribution. Specifically, pH was positively correlated with orders such as *Hypocreales* and *Sordariales*, while TOC, TN, conductivity, and Cl^−^ were closely associated with orders such as *Agaricales* and *Thelebolales*. As verified by 999 Monte Carlo permutation tests, the effects of the main influencing factors (e.g., pH, Cl^−^, and Na^+^) on fungal community composition were statistically significant (*p* < 0.05).

## 4. Discussion

### 4.1. Niche Differentiation Across Different Soil Types and Its Regulation of Microbial Community Structure

The differences in physicochemical properties among brown soil (forestland), black soil, and meadow soil (both cultivated lands) (Table 1) directly reflect the long-term impacts of contrasting land-use regimes. Prolonged forest cover facilitates the accumulation and humification of organic matter, driven primarily by continuous litter input and minimal anthropogenic disturbance, thereby resulting in elevated TOC and TN levels, alongside a relatively acidic soil environment [38]. RDA (Figure 10 and Figure 11) identified TOC and pH as the dominant environmental factors governing the spatial differentiation of bacterial community structures [39]. Under such habitat conditions, high TOC availability combined with acidic conditions collectively selects for K-strategists that are adapted to low nutrient availability, consistent with a typical K-selection environment characterized by stable resource supply but intense interspecific competition. In contrast, black soil and meadow soil, as arable lands, have undergone substantial habitat modification (e.g., frequent tillage), which disrupts soil aggregate structure and accelerates oxidative decomposition of organic matter. Concurrently, crop harvesting results in a net carbon output from the soil pool. Although fertilization partially replenishes nutrients, these systems generally exhibit a high-disturbance, pulsed-resource input regime [40]. RDA further reveals that this environment is marked by higher pH and lower organic carbon content, conditions that are more conducive to rapid utilization of intermittent nutrient pulses by r-strategists (copiotrophic groups). Collectively, these findings demonstrate that environmental filtering, driven by the synergistic interplay between pH and carbon availability, constitutes a key pathway underlying the divergence of microbial ecological strategies across different soil types.

The distinct niches shaped by different soil types markedly drove the differentiation of microbial beta diversity. NMDS analysis (Figure 5) revealed that the brown soil community was clearly separated from the black and meadow soil communities along the first axis. This separation is attributable to the long-term forest cover of the brown soil, in contrast to the cultivated management of the other two soils. Soil type thus emerged as a key predictor governing the systematic restructuring of microbial community composition. This overarching pattern provides a critical foundation for subsequent analyses of ecological strategy differentiation within the community.

### 4.2. Mechanisms Driving Ecological Strategy Differentiation in Microbial Communities

Through in-depth taxonomic profiling of soil microbial communities, this study demonstrates that functional differentiation across habitats is not merely a result of species presence or absence [41] but is primarily driven by shifts in the ecological strategies adopted by the dominant taxa. This finding provides a critical theoretical basis for elucidating the internal regulatory mechanisms governing soil functional potential [42,43].

In the long-term stable yet resource-simple environment of brown soil, the microbial community exhibits pronounced K-strategy traits, characterized by slow growth, efficient resource utilization, and strong capacity to degrade recalcitrant organic matter, thereby occupying specific ecological niches. The core of this strategy is reflected in the adaptive specialization of dominant bacterial and fungal taxa: *Mortierellomycota*, a typical saprophytic fungus in forest soils, predominates in the fungal community (Figure 7 and Figure 8). This group acts as a key functional guild driving the transformation of complex litter and root exudates, and directly participates in the central processes of organic matter cycling in forest ecosystems [24,44]. Furthermore, the marked enrichment of Acidobacteriota and Chloroflexi within the bacterial community further reinforced this oligotrophic adaptive strategy. As a representative oligotrophic phylum, *Acidobacteria* exhibit competitive advantages in low-pH, nutrient-limited soils and contribute to the degradation of complex carbohydrates [45]. The order *Acidobacteria* comprises typical oligotrophic bacteria that possess a competitive advantage in soils with low pH and limited nutrient availability. Their genomes encode a diverse array of carbohydrate-active enzymes capable of efficiently degrading complex plant-derived polysaccharides—such as cellulose and xylan; this may be a key factor contributing to their dominance in forest soils [46]. The synergistic interactions among these dominant taxa collectively establish a tightly coupled nutrient cycling system that efficiently drives the slow decomposition of recalcitrant organic matter in forest ecosystems.

In contrast to the oligotrophic-adapted communities in brown soil, agricultural management of black and meadow soils has selected for r-strategist-dominated assemblages that prioritize rapid resource acquisition, driven by frequent tillage and fertilization. These microorganisms exhibit rapid growth and reproductive responses to pulse-type resource inputs, such as crop residues and root exudates, demonstrating high adaptability to nutrient-rich (eutrophic) conditions. This opportunistic strategy is particularly evident in the bacterial community composition. The high abundance of *Proteobacteria* and *Bacteroidota* is a typical indicator of eutrophic environments [39,47]. Among these, the *Proteobacteria* (particularly, the *α-* and *β-Proteobacteria* subclasses) are typical r-strategists that respond rapidly to newly introduced organic matter; the *Bacteroidetes*, owing to their genomes encoding a large repertoire of glycoside hydrolases, have become important degraders of plant-derived hemicellulose and cellulose, playing a crucial role in agricultural practices such as straw incorporation into the soil; these two bacterial phyla are widely recognized as typical eutrophic bacteria that respond swiftly to newly added organic matter [32]. Likewise, the fungal community exhibits distinct r-strategy traits. *Sordariaceae* and *Hypocreaceae*, which are enriched in agricultural soils, act as early colonizers of fresh plant residues and manure. Their short life cycles and rapid reproductive capacities make them well adapted to the high-turnover resource dynamics characteristic of agroecosystems [48,49,50].

### 4.3. Multi-Dimensional Driving Mechanisms and Management Implications of Soil Microbial Ecological Strategy Differentiation

Redundancy analysis (RDA) (Figure 10 and Figure 11) identified total organic carbon (TOC) [51,52] and pH [53,54] as the two core environmental drivers governing the differentiation of microbial ecological strategies. However, their regulatory influences are not independent but rather operate through a complex synergistic effect involving carbon quantity, carbon quality, and the local microenvironment. The impact of TOC depends not only on its total content but also on its quality attributes [55]. The brown soil is mostly covered by woodland. Although the TOC content is high, the high TOC content may be mainly composed of stubborn components such as lignin and humus [56,57]. This low-quality carbon source provides a stable niche advantage for K-strategists (e.g., *Acidobacteria*) that possess enzymatic machinery for degrading complex organic matter. In contrast, although black and meadow soils exhibit relatively lower total TOC, frequent crop rotations introduce substantial inputs of fresh, readily decomposable plant residues, and this high-quality carbon source greatly promotes the rapid proliferation of r-strategists [58]. Meanwhile, pH acts as a stringent environmental filter, exerting pronounced selective pressure on microbial communities [59]. Although the overall pH range across the Hailun site is relatively narrow, with all values falling within the weakly acidic spectrum, the more acidic conditions in brown soil (pH = 5.75) impose a strong directional selection favoring acid-tolerant groups such as *Acidobacteria* [60] and *Mortierella*. Conversely, the pH range of the cultivated soils (5.98–6.19) creates a more favorable microenvironment for eutrophic-preferring bacteria (e.g., *Bacteroidetes* [61]) and fungi (such as *Fecalibacter*), thereby facilitating their dominance under agricultural management [62].

Although no macroscopic salinization was observed in the soil (conductivity < 0.19 mS/cm), redundancy analysis (RDA) revealed that Cl^−^ and Na^+^ exerted significant effects on microbial community structure, suggesting that residual trace salts may impose long-term cryptic selection on the community through sustained osmotic stress [63]. First, Na^+^, as a key ion for osmotic regulation, can produce continuous osmotic stress on microbial cells even at low and medium concentrations. Second, Cl^−^, as an essential trace element in organisms, participates in the regulation of charge balance and enzyme activity in cells, but small fluctuations in its concentration can change the potential difference of the cell membrane, which in turn affects the efficiency of transmembrane transport of nutrients [64]. Notably, agricultural activities (especially irrigation and tillage) may further amplify the ecological effects of Na^+^ and Cl^−^ by changing the dynamics of soil water and salt transport [65]. The evaporation and concentration of irrigation water can lead to surface salt accumulation.

In summary, this study elucidates the underlying mechanisms by which agricultural reclamation drives a functional paradigm shift in the microbial communities of black soils. In brown soil, the combination of complex recalcitrant carbon sources, an acidic microenvironment, and mineral-mediated adaptive filtering sustains a microbial system dominated by K-strategists, with a functional inclination toward carbon sequestration. In contrast, black and meadow soils subjected to agricultural management have substantially promoted a transition toward r-strategist dominance and a functional bias toward carbon mineralization [66,67]. This shift is driven by the introduction of readily decomposable carbon sources, elevated soil pH, and altered water-salt regimes, which collectively accelerate soil organic matter decomposition and exacerbate the degradation of black soil resources [68,69]. Therefore, current agricultural management practices in the Hailun region should prioritize enhancing soil carbon pool stability. This can be achieved through the application of stable organic amendments, such as compost and biochar, which provide substrates for K-strategist microorganisms. Concurrently, conservation tillage measures, including no-tillage and mulching, should be implemented to minimize physical disturbance of soil structure and preserve the microhabitats essential for oligotrophic taxa [70]. Furthermore, irrigation systems should be optimized to mitigate the risk of secondary salinization, thereby reducing persistent ionic stress and its disruptive effects on microbial community functioning.

### 4.4. Limitations and Future Perspectives

This study aims to reveal the differentiation pattern and driving mechanism of microbial communities under different soil types in the typical black soil area of the Songnen Plain, but the research design itself still has limitations. This study has two principal limitations. First, 16S rRNA and ITS amplicon sequencing resolves community structure but not functional traits (e.g., enzyme activities and substrate utilization); hence, the inferred r/K strategies are indirect deductions based on taxon relative abundances and require functional validation via metatranscriptomics or metaproteomics. Second, only bulk TOC and TN were measured, without fractionating carbon components (lignin, humus, and particulate organic C); thus, the suggestion that high TOC in brown soil is dominated by recalcitrant carbon remains speculative and awaits chemical fractionation.

Despite these constraints, our regional-scale systematic sampling and multivariate analyses first reveal microbial community differentiation patterns among soil types in the Songnen Plain black soil area and then introduce the r/K framework into a preliminary mechanistic discussion. These findings offer a theoretical basis for sustainable soil management and lay the groundwork for subsequent mechanism-oriented studies.

## 5. Conclusions

This study investigated typical cultivated black soil and meadow soil in Hailun City (Songnen Plain, China), along with adjacent primary forest brown soil, to elucidate how land-use patterns drive microbial ecological strategy differentiation. Soil type emerged as a key determinant of physicochemical properties and community structure: cultivated soils exhibited significantly lower TOC and higher pH than forest soil. These environmental contrasts drove clear community segregation along the r/K axis—brown soil, dominated by *Mortierellomycota* and *Acidobacteriota*, represented a K-strategist community specialized in recalcitrant organic matter decomposition and carbon sequestration, whereas black and meadow soils, enriched with *Proteobacteria*, *Bacteroidota*, and *Sordariaceae*, constituted r-strategist communities adept at rapid labile carbon utilization and carbon release. TOC bioavailability and pH were identified as core environmental filters governing competitive advantage between the two strategies. Given the cold, dry local climate, conservation tillage, such as no-till or deep tillage at 30–35 cm with straw mulching, is recommended to minimize aggregate disruption and organic matter exposure. However, such practices require long-term implementation (≥5 years) to significantly alter the r/K ratio, whereas short-term (1–2 years) effects are likely negligible. These findings provide a theoretical basis for sustainable soil management and a foundation for further mechanistic studies.

## Figures and Tables

**Table 1:** Physical and chemical properties of different types of soil in Hailun.

Soil Properties	Soil Type		
	Brown	Meadow	Black
Na^+^ (g/kg)	9.95 ± 1.25 a	10.39 ± 0.69 a	10.05 ± 0.64 a
K^+^ (g/kg)	20.79 ± 1.73 a	21.31 ± 0.61 a	21.46 ± 0.84 a
TN (g/kg)	3.78 ± 2.61 a	2.59 ± 0.61 b	2.46 ± 0.51 a
Cl^−^ (g/kg)	0.09 ± 0.02 a	0.08 ± 0.01 b	0.07 ± 0.01 a
TOC (g/kg)	4.26 ± 3.45 a	2.95 ± 0.69 b	2.84 ± 0.66 a
Conductivity (ms/cm)	0.08 ± 0.05 a	0.06 ± 0.03 a	0.06 ± 0.02 a
pH	5.75 ± 0.97 a	6.19 ± 0.69 a	5.98 ± 0.51 a

Note: Values are mean ± SD. Different lowercase letters in the same row indicate significant differences among stand types (ANOVA, *p* < 0.05, Tukey’s HSD post hoc test).

## Data Availability

The original contributions presented in this study are included in the article. Further inquiries can be directed to the corresponding author.

## References

[1] Hughes J.B., Hellmann J.J., Ricketts T.H., Bohannan B.J.M. (2001). Counting the Uncountable: Statistical Approaches to Estimating Microbial Diversity. Appl. Environ. Microbiol..

[2] Zhu Y.G., Peng J.J., Wei Z., Shen Q.R., Zhang F.S. (2021). Linking the soil microbiome to soil health. Sci. Sin. Vitae.

[3] Si P., Shao W., Yu H., Yang X., Gao D., Qiao X., Wang Z., Wu G. (2018). Rhizosphere Microenvironments of Eight Common Deciduous Fruit Trees Were Shaped by Microbes in Northern China. Front. Microbiol..

[4] Schadt C.W. (2010). Soil microbial community responses to multiple experimental climate change drivers. Appl. Environ. Microbiol..

[5] Maestre F.T., Delgado-Baquerizo M., Jeffries T.C., Eldridge D.J., Singh B.K. (2015). Increasing aridity reduces soil microbial diversity and abundance in global drylands. Proc. Natl. Acad. Sci. USA.

[6] Barberán A., Bates S.T., Casamayor E.O., Fierer N. (2012). Using network analysis to explore co-occurrence patterns in soil microbial communities. ISME J..

[7] Banerjee S., Schlaeppi K., Van Der Heijden M.G.A. (2018). Keystone taxa as drivers of microbiome structure and functioning. Nat. Rev. Microbiol..

[8] Bardgett R.D., Putten W.H.V.D. (2014). Belowground biodiversity and ecosystem functioning. Nature.

[9] Moras S., Mazor M., Amit I., Gerth P., Trautwein-Schult A., Maa S., Grinshpan I., Shelly Y., Levin L., Becher D. (2026). Community context reshapes microbial proteomes and reduces functional overlap. Nat. Microbiol..

[10] Bakkeren E., Piskovsky V., Lee M.N.Y., Jahn M.T., Foster K.R. (2025). Strain displacement in microbiomes via ecological competition. Nat. Microbiol..

[11] Jiang Y.F., Guo X., Sun K., Rao L., Li W.F. (2017). Spatial Variability of C-to-N Ratio of Farmland Soil in Jiangxi Province. Environ. Sci..

[12] Chen D., Mi J., Chu P., Cheng J., Zhang L., Pan Q., Xie Y., Bai Y. (2015). Patterns and drivers of soil microbial communities along a precipitation gradient on the Mongolian Plateau. Landsc. Ecol..

[13] Lyu L., Wang C., Fan K., Li J., Yang T., Gao G., Sun R., Wang J., Xu X., Zhang Y. (2025). Microbial life-history strategies mediate temperature effects on organic carbon pools in black soils. Soil Ecol. Lett..

[14] Fang H.R., Liu J.J., Chen X.L., Jiang Y., Liu X.Z., Gu H.D., Wan S.M., Xiao Y. (2024). Effects of long-term combined application of organic and chemical fertilizers on bacterial community characteristics and soybean yields. Chin. J. Eco-Agric..

[15] Zhang M., Bai Z., Zhang W., Feng H.M., Wu Y.Y., Ding X.L., Zhang X.D. (2007). Effects of long-term fertilization on r-K strategy microbial populations in farmland mollisol. Chin. J. Ecol..

[16] Guo Y., Chen X., Wu Y., Zhang L., Cheng J., Wei G., Lin Y. (2018). Natural revegetation of a semiarid habitat alters taxonomic and functional diversity of soil microbial communities. Sci. Total Environ..

[17] Wang G.H., Hu X.J., Yu Z.H., Chen L.X., Liu J.J. (2024). Effects of fertilization on soil microbial community diversity in Chinese arable black soils: Research progress and prospects. Soils Crops.

[18] Wei W., Xu Y.L., Zhu L., Han X.Z. (2013). Effect of long-term fertilization on soil microbial communities in farmland of black soil. Acta Pedol. Sin..

[19] Yang B., Feng W., Zhou W., He K., Yang Z. (2024). Association between Soil Physicochemical Properties and Bacterial Community Structure in Diverse Forest Ecosystems. Microorganisms.

[20] Song Y.H., Liu K., Dai H.M., Xu J., Zhang Z.H., Liang S. (2022). Palynological assemblages of typical black soil profile in the eastern Songliao Plain and their age and its implication for paleoclimatic. Geol. Bull. China.

[21] Huang X.R., Shun Z.C., Hu X.N., Chen H.F., Chen G. (2024). Vertical Changes of Quaternary Sediment Microbial Communities in the South Bank of Laizhou Bay. J. Univ. Jinan (Sci. Technol.).

[22] Godfray H.C., Beddington J.R., Crute I.R., Haddad L., Lawrence D., Muir J.F., Pretty J., Robinson S., Thomas S.M., Toulmin C. (2010). Food security: The challenge of feeding 9 billion people. Science.

[23] Feng Y.J., Zhang W., Chen Q., Ma C.H. (2007). Physico-Chemical Characteristics and Microbial Composition of Saline-Alkaline Soils in Songnen Plain. Soils.

[24] Hu F., Wang F., Han X.Z., Xu M., Fu Y.H., Yan J., Jia Z.J., Tiedje J., Jiang X. (2022). Succession of Microbial Community in Typical Black Soil under Different Land Use Pattern. Soils.

[25] Song Y.H., Zhang Z.H., Yang F.C., Liang S., Wei M.H., Liu K., Xu J. (2020). Vertical distribution of major nutrient elements in typical black soil sections in hailun, heilongjiang province: Before and After Reclamation. Geol. Resour..

[26] Van der Heijde M.G., Bardgett R.D., Van Straalen N.M. (2008). The unseen majority: Soil microbes as drivers of plant diversity and productivity in terrestrial ecosystems. Ecol. Lett..

[27] Liu J., Sui Y., Yu Z., Shi Y., Chu H., Jin J., Liu X., Wang G. (2014). High throughput sequencing analysis of biogeographical distribution of bacterial communities in the black soils of northeast China. Soil Biol. Biochem..

[28] Caporaso J.G., Lauber C.L., Walters W.A., Berg-Lyons D., Lozupone C.A., Turnbaugh P.J., Fierer N., Knight R. (2011). Microbes and Health Sackler Colloquium: Global patterns of 16S rRNA diversity at a depth of millions of sequences per sample. Proc. Natl. Acad. Sci. USA.

[29] Alberto O., Erica L., Henrik N.R., Mariangela G., Alfredo V., Paola B., Valeria B., Mari M. (2012). Unravelling Soil Fungal Communities from Different Mediterranean Land-Use Backgrounds. PLoS ONE.

[30] Bolyen E., Rideout J.R., Dillon M.R., Bokulich N.A., Abnet C.C., Al-Ghalith G.A., Alexander H., Alm E.J., Arumugam M., Asnicar F. (2019). Reproducible, interactive, scalable and extensible microbiome data science using QIIME 2. Nat. Biotechnol..

[31] Fierer N., Bradford M.A., Jackson R.B. (2007). Toward an ecological classification of soil bacteria. Ecology.

[32] Yang Y., Dou Y., Wang B., Xue Z., Wang Y., An S., Chang S.X. (2023). Deciphering factors driving soil microbial life-history strategies in restored grasslands. Imeta.

[33] Muhammad W., Yu X., Wu J., Yang X., Mo Y., Luo Q., Feng L., Pu X., Zhou W., Song L. (2026). A global meta-analysis reveals coupled biogeochemical and microbial successional trajectories in forest soils. For. Ecosyst..

[34] Estrela S., Sánchez Á., Rebolleda-Gómez M. (2021). Multi-Replicated Enrichment Communities as a Model System in Microbial Ecology. Front. Microbiol..

[35] Estrela S., Sanchezgorostiaga A., Vila J.C.C., Sanchez A. (2021). Nutrient dominance governs the assembly of microbial communities in mixed nutrient environments. elife.

[36] Qiu D.C., Liao Z.Y., Xing Q.H., Wang H.S., Liu J., Zhao B.S. (2022). The correlation analyses between bacterial community and the crucial environmental factors in saline-alkali soil of Songnen Plain. Microbiol. China.

[37] Zeng W.A., Yang Z.Y., Huang Y., Gu Y.B., Tao J.M., Liu Y.J., Xie P.F., Cai H.L., Yin H.Q. (2022). Response of soil bacterial community structure and co-occurrence network topology properties to soil physicochemical properties in long-term continuous cropping farmland. Acta Microbiol. Sin..

[38] Wang D., Wang J., Zhang Y., Chen X., Chen J., Shi X. (2025). Nitrogen-Induced Soil Acidification Reduces Soil Carbon Persistence by Shifting Microbial Keystone Taxa and Increasing Calcium Leaching. Agronomy.

[39] Fierer N. (2017). Embracing the unknown: Disentangling the complexities of the soil microbiome. Nat. Rev. Microbiol..

[40] He D., Dai Z., Cheng S., Shen H., Lin J., Zhao K., Rodrigues J.L.M., Kuzyakov Y., Xu J. (2025). Microbial life-history strategies and genomic traits between pristine and cropland soils. mSystems.

[41] Chen Y.L., Ding J.Z., Peng Y.F., Li F., Yang G.B., Liu L., Qin S.Q., Fang K., Yang Y.H. (2016). Patterns and drivers of soil microbial communities in Tibetan alpine and global terrestrial ecosystems. J. Biogeogr..

[42] Bittleston L.S., Gralka M., Leventhal G.E., Mizrahi I., Cordero O.X. (2020). Context-dependent dynamics lead to the assembly of functionally distinct microbial communities. Nat. Commun..

[43] Hou D.Y., Li A.X., Lv S.L., Dong J.Y., Chen N., Wang X. (2025). Analysis of phytoplankton community structure and its relationship with environmental factors in coastal waters of eastern Leizhou Peninsula during spring and autumn. South China Fish. Sci..

[44] Xie K., Sun T., Miu Y., Li Y., Xu Y., Cheng Y., Lu X. (2026). Variation in Soil Microbial Communities Across Plantation Types in the Yellow River Floodplain of Western Shandong, China. Microorganisms.

[45] Kielak A.M., Barreto C.C., Kowalchuk G.A., Van Veen J.A., Kuramae E.E. (2016). The Ecology of Acidobacteria: Moving beyond Genes and Genomes. Front. Microbiol..

[46] Islam Z.F., Cordero P.R.F., Feng J., Chen Y.J., Bay S.K., Jirapanjawat T., Gleadow R.M., Carere C.R., Stott M.B., Chiri E. (2019). Two Chloroflexi classes independently evolved the ability to persist on atmospheric hydrogen and carbon monoxide. ISME J..

[47] Kruczyńska A., Kuniar A., Podlewski J., Somczewski A., Grzdziel J., Marzec-Grzdziel A., Gazka A., Wolińska A. (2023). Bacteroidota structure in the face of varying agricultural practices as an important indicator of soil quality—A culture independent approach. Agric. Ecosyst. Environ..

[48] Handa I.T., Aerts R., Berendse F., Berg M.P., Bruder A., Butenschoen O., Chauvet E., Gessner M.O., Jabiol J., Makkonen M. (2014). Consequences of biodiversity loss for litter decomposition across biomes. Nature.

[49] Vo?í?ková J., Baldrian P. (2013). Fungal community on decomposing leaf litter undergoes rapid successional changes. ISME J..

[50] Wang S., Cheng J., Li T., Liao Y. (2020). Response of soil fungal communities to continuous cropping of flue-cured tobacco. Sci. Rep..

[51] Cheng S., Ju X., Wang K., Zhang W., Gu C. (2026). Rate-Dependent Effects of Biochar on Soil Fertility and Bacterial–Fungal Communities in Maize Fields of the Black Soil Region: A Three-Year Field Study. Microorganisms.

[52] Hu Y., Xiang D., Veresoglou S.D., Chen F., Chen Y., Hao Z., Zhang X., Chen B. (2014). Soil organic carbon and soil structure are driving microbial abundance and community composition across the arid and semi-arid grasslands in northern China. Soil Biol. Biochem..

[53] Rousk J., Bååth E., Brookes P.C., Lauber C.L., Lozupone C., Caporaso J.G., Knight R., Fierer N. (2010). Soil bacterial and fungal communities across a pH gradient in an arable soil. ISME J..

[54] Lu L., Luo X., Xu J.F., Dong S.Q., Liang S.S., Xu L.F., Liu L.Z., Li Y.H. (2025). Effects of pH and hydrochemical types on archaeal community structures in salt lakes of Badain Jaran Desert, Inner Mongolia. Microbiol. China.

[55] Rutigliano F.A., D’Ascoli R., De Santo A.V. (2004). Soil microbial metabolism and nutrient status in a Mediterranean area as affected by plant cover. Soil Biol. Biochem..

[56] Nie X., Wang D., Yang L., Zhou G. (2021). Controls on variation of soil organic carbon concentration in the shrublands of the north-eastern Tibetan Plateau. Eur. J. Soil Sci..

[57] Yang Y., Chen J., Zheng Y., Jiang R., Sang Y., Zhang J. (2024). The Effects of Mixed Robinia pseudoacacia and Quercus variabilis Plantation on Soil Bacterial Community Structure and Nitrogen-Cycling Gene Abundance in the Southern Taihang Mountain Foothills. Microorganisms.

[58] Kong D., Liu N., Guan Y., Ren C., Sun J., Guo C., Ren G., Feng Y. (2026). Soil Fungal Community Dynamics Are More Strongly Influenced by Crop Growth Stage than by Straw Retention Amount Under Long-Term Wheat–Soybean Rotation. Microorganisms.

[59] Lauber C.L., Hamady M., Knight R., Fierer N. (2009). Pyrosequencing-Based Assessment of Soil pH as a Predictor of Soil Bacterial Community Structure at the Continental Scale. Appl. Environ. Microbiol..

[60] Jiao F., Qian L., Wu J., Zhang D., Zhang J., Wang M., Sui X., Zhang X. (2024). Diversity and Composition of Soil Acidobacterial Communities in Different Temperate Forest Types of Northeast China. Microorganisms.

[61] Pan X., Raaijmakers J., Carrión V. (2023). Importance of Bacteroidetes in host-microbe interactions and ecosystem functioning. Trends Microbiol..

[62] Li Z., Gao X., Li D., Gao J., Li J., Cai Z., Sun N., Wu L., Xu M. (2010). Differential responses of bacteria, fungi, and C, N, P functions to soil acidification in farmland: A meta-analysis. Environ. Technol. Innov..

[63] Apte S.K., Thomas J. (1997). Possible amelioration of coastal soil salinity using halotolerant nitrogen-fixing cyanobacteria. Plant Soil.

[64] White P.J., Broadley M.R. (2001). Chloride in Soils and its Uptake and Movement within the Plant: A Review. Ann. Bot..

[65] Hui R., Tan H. (2024). Divergence of nutrients, salt accumulation, bacterial community structure and diversity in soil after 8 years of flood irrigation with surface water and groundwater. BMC Microbiol..

[66] Qian R., Gao L., Liu J., Biswas A., Peng X. (2026). Linking microbial life strategies to carbon mineralization under diverse tillage practices: Insights from eroding black soil hillslopes. Agric. Ecosyst. Environ..

[67] Jiamin X., Haonan W., Chao X., Yajun H., Zhiwen X., Dongxu Z., Abo L., Xiaomeng W., Tida G., Gehong W. (2025). The distribution of aerobic bacteria in Chinese cropland is linked to the soil texture. Front. Microbiol..

[68] Sui Y.Y., Jiao X.G., Gao C.S., Cheng W., Zhang X.Y., Liu X.B. (2009). The Relationship among Organic Matter Content and Soil Microbial Biomass and Soil Enzyme Activities. Chin. J. Soil Sci..

[69] Pan Z., Xie X.J., Li Q.H., Gan Z.Y., Hu T., Yang J., Deng Y.M., Gan Y.Q., Zhang Y.P. (2022). Microbial Community Structure and Its Response to Environment in Mangrove Sediments of Dongzhai Port. Earth Sci..

[70] Na L., Liu Y., Nan Q., Chen L., Dong D., Wu W., Tang J., Yang S., Liu Y. (2026). Microbial life-history strategies mediate differential effects of straw and biochar amendments on soil POC/MAOC dynamics and SOC sequestration. Biochar.

